# Energy-Saving LED Light Affects the Efficiency of the Photosynthetic Apparatus and Carbohydrate Content in *Gerbera jamesonii* Bolus ex Hook. f. Axillary Shoots Multiplied In Vitro

**DOI:** 10.3390/biology10101035

**Published:** 2021-10-12

**Authors:** Monika Cioć, Krzysztof Tokarz, Michał Dziurka, Bożena Pawłowska

**Affiliations:** 1 Department of Ornamental Plants and Garden Art, Faculty of Biotechnology and Horticulture, University of Agriculture in Kraków, 29 Listopada 54, 31-425 Kraków, Poland; bozena.pawlowska@urk.edu.pl; 2 Department of Botany, Physiology and Plant Protection, Faculty of Biotechnology and Horticulture, University of Agriculture in Kraków, 29 Listopada 54, 31-425 Kraków, Poland; krzysztof.tokarz@urk.edu.pl; 3 Department of Developmental Biology, The Franciszek Górski Institute of Plant Physiology, Polish Academy of Sciences, Niezapominajek 21, 30-239 Kraków, Poland; m.dziurka@ifr-pan.edu.pl

**Keywords:** LED light, PS II, photosystem II, carbohydrates, light emitting diodes, gerbera, HPLC, soluble sugars

## Abstract

**Simple Summary:**

Gerbera is one of the most important ornamental plants on the cut flower market. The basic reproduction methods of numerous cultivars of this species are in vitro techniques. In in vitro cultures, all plant growth conditions are controlled, including the light (intensity, quality, and duration). In tissue cultures, light quality is the most important factor that influences plant morphogenesis (growth and development). Light emitting diodes (LEDs), in contrast to the commonly used fluorescent lamps, allow for adjusting the light quality to the specific requirements of plants. LEDs are also energy-efficient and contain no harmful substances (e.g., mercury). The aim of the study was to analyze the effect of different light qualities emitted by LEDs during in vitro multiplication of *Gerbera* on its metabolic and physiological development. We compared endogenous carbohydrate content in the tissues and the condition of the photosynthetic apparatus in plants grown under fluorescent lamps and LED light. The study showed that the use of LEDs did not disturb the secondary metabolism of carbohydrates and the multiplied shoots were of high quality. The mixture of red and blue LED light in a 7:3 proportion is recommended for gerbera micropropagation. This light quality positively influenced the functioning of the photosynthetic apparatus.

**Abstract:**

An energy-saving light emitting diode (LED) system allows for adjustment of light quality, which affects plant development and metabolic processes in in vitro cultures. The study investigated the content of endogenous carbohydrates and the condition of the photosynthetic apparatus of *Gerbera jamesonii* Bolus ex Hook. f. Our aim was to analyze the effects of different LED light qualities—100% red light (R LED), 100% blue (B LED), a mixture of red and blue (7:3) (RB LED), and a fluorescent lamp as a control (Fl)—during the multiplication of axillary shoots. After 40 days, the culture measurements were performed using a non-invasive pulse amplitude modulation (PAM) fluorimeter. Sugar content was assessed with high performance liquid chromatography (HPLC). Two forms of free monosaccharides (glucose and fructose), two sugar alcohol derivatives (inositol and glycerol), and seven forms of free oligosaccharides were identified. Of those, glucose content was the highest. LEDs did not disturb the sugar metabolism in multiplied shoots. Their monosaccharides were three times more abundant than oligosaccharides; the same results were found in plants grown under control light. R light depleted the performance of the photosynthetic apparatus and caused its permanent damage. The RB LED spectrum ensured the most efficient non-photochemical quenching of the photosystem II (PS II) excitation state and high shoot quality.

## 1. Introduction

Gerbera is one of the most important ornamental plants on the cut flower market. In some countries it is potted, and a garden plant is cultivated in flower beds and in container compositions. The basic reproduction methods of numerous cultivars of this species are in vitro techniques that provide microcuttings for horticultural production [1,2]. For practical reasons, the micropropagation needs to be not only efficient but it must also offer high quality shoots. Appropriate propagation ensures success at the subsequent stages of clonal reproduction, from in vitro rooting and acclimatization to a ready microcutting, which determines the success of further production [3]. During micropropagation in in vitro laboratories, fluorescent lamps with a constant spectrum are normally used as a light source, without the possibility of adjusting light quality [4]. Only the use of solid state lighting light emitting diode (SSL LED) technology makes it possible to select specific qualitative lighting conditions, appropriate for specific development processes or for each genotype [5,6]. In addition, the light emitting diode system is an environmentally friendly solution with lower weight and volume and reduced heat emission. The economic aspect is also essential, as LED lamps feature significantly lower electricity consumption and higher energy efficiency than those of fluorescent lamps [5,7,8,9]. LEDs are also safer for the environment during disposal because they do not contain harmful mercury [10].

Studies on gerbera micropropagation under LEDs are already available [9,11,12,13]. They show that this light in in vitro cultures of gerbera allows for controlling morphogenesis and may even increase the productivity of multiplication and rooting of axillary shoots. This is interesting if the quality and functionality of the produced plants are also high. These questions can be answered with basic research studies that analyze the plant processes initiated by a light stimulus.

Light may sometimes be a form of abiotic stress. Signaling and dynamics of changes in the level of soluble sugars are crucial for controlling organogenesis and for management of biotic and abiotic stresses [14]. Plant defense response may be manifested by an increased production of secondary metabolites, including carbohydrates [15]. Light can affect the level of endogenous plant sugars. Its quality, in comparison with the intensity or photoperiod, exerts a much more complex influence on plant growth and development in vitro [16].

The source of organic carbon in in vitro cultures are sugars added to the medium. Endogenous sugars determine shape and modify plant metabolism. They provide and store energy, participate in transport and organ formation, and are the donors of carbon skeletons and respiratory substrates [17,18]. By translating their nutrient status to transcriptional regulation, plants can modulate their growth both at the systemic and local level. They do that by using the patterns specific for individual tissues or cells and by coordinating developmental paths with available carbohydrates [18]. Sugars are also included in DNA and RNA and may modify some proteins [19,20]. Additionally, together with growth regulators, they participate in signal transduction [21,22]. Their interactions with phytohormones control metabolic processes at various stages of plant development and in response to stress [17]. These processes can be modified depending on the type of carbohydrate. For example, sucrose, which is usually added to in vitro media, is the major transporting sugar in plants, and can be sensed directly or through its cleavage products [18]. Availability of carbohydrate substrates is required for the growth of plant tissues. In addition, functioning of meristematic cells depends on the nutritional status of the plant due to a high demand for sugars during cell division and differentiation [23].

Light is a fundamental element enabling photosynthetic activity of plants. It also represents the essential substrate in the physico-chemical transformation phase as the primary source of energy [24]. In in vitro cultures, photosynthesis is very limited due to environmental conditions [25,26]. The presence of exogenous sugars limits photosynthesis and impairs proper development of the photosynthetic apparatus [27,28,29]. Assimilation of external carbon dioxide is rarely observed due to the availability of organic carbon in the medium, as well as 100% humidity and very limited diffusion. Plants cultivated under such conditions are much more often characterized by a mixed, i.e., photoautotrophic/heterotrophic, nutrition pattern [30]. Light naturally affects the photosynthetic apparatus and the possibility of effective photoautotrophy in plants [31]. Both the visible and near-infrared spectra are crucial for the formation of a well-functioning photosynthetic apparatus [32]. The efficiency of photosynthesis depends on the sequence of metabolic processes during light and dark reactions, the activity of enzymes involved in carbon assimilation, the structure of the photosynthetic apparatus, and the transport of photosynthetic intermediates between cellular compartments [33]. The quality of light affects the photosynthesis, e.g., by its influence on the chloroplast structure, regulation of leaf size, opening of the stomata, or photosystem II (PS II) [34]. A stress caused by an abiotic factor, e.g., light, may damage the photosynthetic apparatus, especially PS II. This damage may trigger the production of harmful reactive oxygen species (ROS), excessive absorption of sunlight by the light-harvesting complex (and excessive stimulation of PS II), photoinhibition, imbalance in the photosynthetic redox signaling pathways, and inhibition of PS II repair [35]. Plants manage the effects of abiotic stress to some extent by dispersing the absorbed energy in nonradiative processes. This is done by reducing the rate of electron transport or converting excessive, absorbed light into thermal energy [36,37,38]. The dissipation of excess excitation energy as heat is known as non-photochemical quenching of chlorophyll fluorescence [35]. The efficiency of photosynthesis in vitro may be affected by high sugar content in the medium that results in the repression of the final products of some photosynthetic enzymes [39,40,41].

Chlorophyll fluorescence, a form of energy dissipation, is a sensitive marker of plant photosynthesis, widely used in recent years and in in vitro cultures. The non-invasive measurement of photosynthesis by means of chlorophyll fluorimetry can provide information on the plant viability and the efficiency of its photosynthetic apparatus [36,42]. The performance of the photosynthetic apparatus determines the success of further in vitro stages, mainly acclimatization [41]. Effective acclimatization and proper further growth may be related to the reconstruction of the photosynthetic apparatus and its performance during multiplication of axillary shoots. These processes may be controlled by the use of a specific light quality applied during in vitro culture [33,43].

The aim of the study was to analyze the effects of different light qualities on the content of endogenous carbohydrates in plant tissues and the condition of the leaf photosynthetic apparatus assessed by chlorophyll fluorescence. The impact was investigated during multiplication of axillary shoots of gerbera. Various light spectra were provided by light emitting diodes (LED).

## 2. Materials and Methods

### 2.1. Plant Material and Growth Conditions

**Figure 1:**
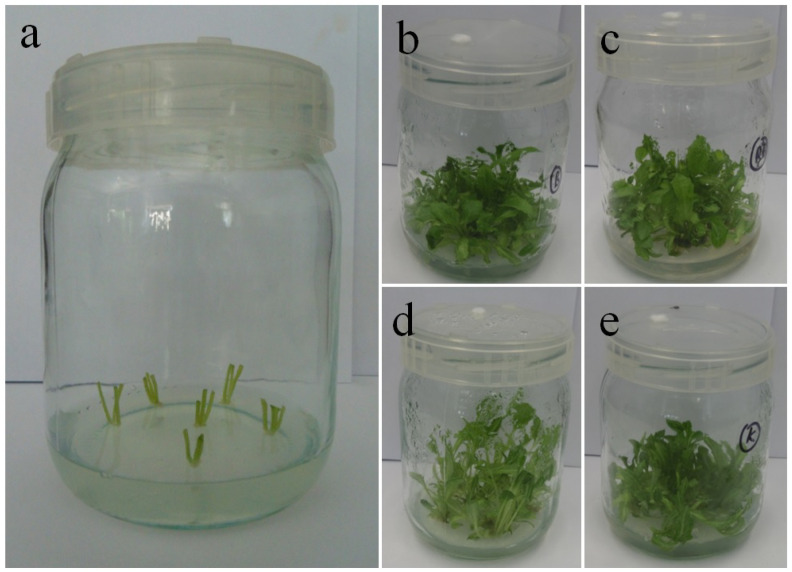
Glass jars (500 mL) covered with polyvinyl chloride caps with an air orifice. (**a**) Explants of gerbera axillary shoots ready for multiplication under different light qualities, (**b**–**e**) multiplied gerberas after 40 days of culture under: (**b**) B LED, (**c**) RB LED, (**d**) R LED and (**e**) control light of Fl.

The study investigated axillary shoots of *Gerbera jamesonii* Bolus ex Hook. f. ‘Big Apple’ cultured in vitro for 40 days under light of different quality. The plant material came from the in vitro plant collection of the Department of Ornamental Plants and Garden Art of the University of Agriculture in Kraków (Kraków, Poland). It is a cultivar selected by Schreurs company (De Kwakel, The Netherlands). We used Murashige and Skoog (MS) growth medium [44] enriched with 30 g/L sucrose, 0.5 µM 1-naphthalene acetic acid (NAA), and 5 µM 6-benzyladenine (BA), solidified with 0.5% BioAgar (Biocorp, Warsaw, Poland), at pH 5.7. The culture was conducted in 50 mL glass jars (Figure 1) and covered with polyvinyl chloride caps with an air orifice (MZ Forma, Łódź, Poland). Using a BTS256 spectrometer (Gigahertz-Optik, Türkenfeld, Germany) and a LI-250A light meter equipped with a Q 50,604 sensor (LI-COR, Lincoln, NE, USA) we set different light qualities emitted by a solid state lighting light emitting diodes system (SSL LED) (PXM, Podłęże, Poland) [12]: 100% red light (670 nm) (R LED), 100% blue light (430 nm) (B LED), and a mixture of red and blue light (7:3) (RB LED). The control was white light provided by a fluorescent (Fl) lamp (Philips TL-D 36W/54). The spectra of the lamps are available in our previous manuscript [12]. For all tested light qualities, the photosynthetic photon flux density (PPFD) was 35 µmol m^−2^ s^−1^. External conditions in the phytotron were as follows: 16/8 h photoperiod (day/night), temperature 23/21 ± 1 °C (day/night), and 80% relative humidity. The culture of multiplied shoots involved five repetitions per treatment, with five explants each (100 explants in total).

### 2.2. Data Collection

When shoot multiplication was finished (40 days), we measured leaf chlorophyll fluorescence and tissue level of endogenous sugars.

The photosystem analysis involved 2140 measurements (535 for each light quality) conducted with a Photosynthesis Yield Analyzer (MINI-PAM, Waltz, Germany). The measurements were carried out on leaf blades from the central whorl of the rosette. The performance of the PS II and chlorophyll fluorescence were assessed with a non-invasive fluorometric method. Chlorophyll a fluorescence induction curve measurements were completed on in vitro grown leaves. The leaves (*n* = 32) from 16 plants of each combination were adapted to darkness for 20 min. After dark adaptation, the minimum (Fo) and maximum fluorescence (Fm) were obtained before and after application of a saturation pulse (8000 µmol (quanta) m^−2^ s^−1^) for a duration of 0.8 s. The maximum potential photochemical quantum yields of PS II (Fv/Fm), plastoquinone pool (Fv/2), as well as the activity of the oxygen evolving complex (OEC) on the donor side of the PSII (Fv/Fo) were calculated according to Bolhar-Nordenkampf et al. [45], Jiang et al. [46], Kalaji et al. [47], and Tokarz et al. [48]. Quantum photosynthetic yield of PSII (Y(II)), photoprotective non-photochemical quenching (Y(NPQ)), and all other non-photoprotective non-photochemical quenching Y(NO) parameters were evaluated using a quenching tests protocol and calculated as described by Klughammer and Schreiber [49]. The results were recorded and analyzed using WinControl Software (WALZ, Germany).

Sugar content was analyzed in representative samples of 18 leaves in three replicates per treatment. The samples were obtained from shoots and leaves of middle age, and each comprised about 0.5 g FW. We assessed their content of free sugars, total soluble sugars, starch, and the average polymerization degree of soluble oligocarbohydrates in four replicates. The analysis was conducted using a method of Janeczko et al. [50], described and modified by Hura et al. [51]. Prior to the analysis the plant material was frozen in liquid nitrogen, then freeze-dried and homogenized (MM 400, Retsch, Haan, Germany). The subsamples of 20 mg DW were extracted in 1 mL of ultra-pure water (15 min shaking at 30 Hz, MM 400) at an ambient temperature after being heated for 5 min at 80 °C. The supernatant was divided into two portions. The first was diluted with acetonitrile 1:1 (*v*/*v*) and analyzed by HPLC for soluble sugar content. The second was used for fructo-oligosaccharide estimation. Fructo-oligosaccharides were estimated after enzymatic hydrolysis of sugar extract by a mixture of exo-inulase and endo-inulinase (Megazyme, Bray, Ireland). Starch was determined in the pellets remaining after the analysis of soluble sugars using enzymatic hydrolysis (α-amylase followed by amyloglucosidase, both from Sigma-Aldrich, Poznan, Poland), according to Mikuła et al. [52]. The released glucose and fructose or glucose, respectively, were measured by the same HPLC method. HPLC estimation of sugars was performed using an Agilent 1200 binary system (Agilent, Waldbronn, Germany) coupled to an ESA Coulochem II electrochemical detector (ESA, Chelmsford, MA, USA). The sugars were chromatographically separated on an RCX-10; 7 µm; 250 × 4.1 mm column (Hamilton, Reno, NV, USA) in a gradient of 75 mM NaOH and 500 mM sodium acetate in 75 mM NaOH. Pulsed amperometric detection was employed. Further technical details are given by Hura et al. [51]. All chemicals used for the analyses, if not specified otherwise, were of analytical grade supplied by Sigma-Aldrich (Poznań, Poland).

### 2.3. Statistical Analysis

All collected data were exposed to a statistical analysis with the use of Statistica software version 13 (TIBCO Software Inc., Palo Alto, CA, USA). The analysis of variance (ANOVA) was used to test for significance of the effects of the treatments. The data were checked for homogeneity of variance. To separate significantly different means and to provide homogeneous groups for the means (at *p* ≤ 0.05), the Duncan post hoc multiple range test was used.

## 3. Results

### 3.1. Photosynthetic Apparatus

**Figure 2:**
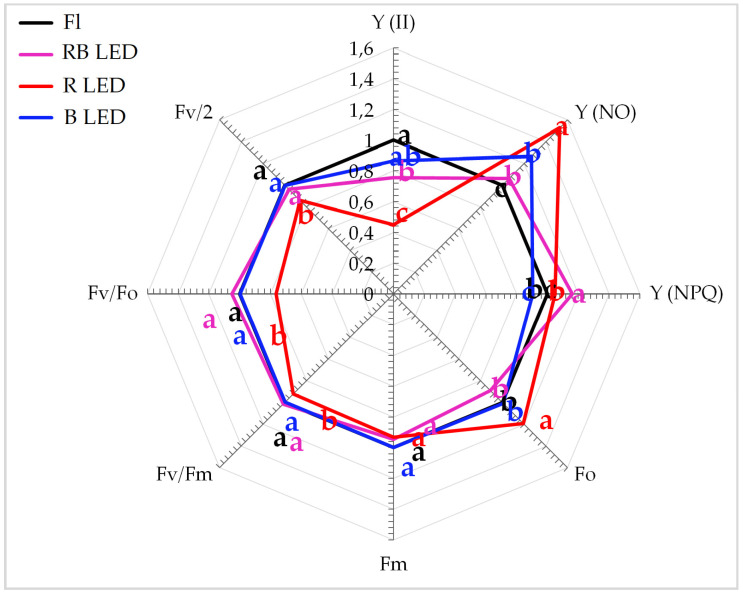
Photosynthetic apparatus efficiency (slow kinetics of Chl a fluorescence parameters) of gerbera plants depending on light source spectral composition after four weeks of culture. Values are given in relation to control (set as 1); Fl—fluorescent lamps (Philips TL-D 36W/54), RB LED—a mixture of red and blue LED light (7:3), R LED—100% red LED light (670 nm), B LED—100% blue LED light (430 nm). The same letters in the chart (a, b, ab) indicate that means are not significantly different according to Duncan’s multiple range test at *p* ≤ 0.05.

The quality of light during plant growth was important for the formation and function of the photosynthetic apparatus. R LED significantly reduced the performance of the photosynthetic apparatus in gerbera leaves. The photosynthetic apparatus was characterized by a significantly lower value of the maximum (potential) yield of PSII (Fv/Fm), activity of the water-splitting complex on the donor side of the PSII (Fv/Fo), and the actual quantum yield of PSII (Y(II)) (Figure 2). Moreover, a significant reduction in the plastoquinone pool as represented by the Fv/2 ratio was noted. At the same time, a significant increase in the danger of permanent damage to PSII (Y(NO)) was observed. The plants treated with B and R LED light combinations were characterized by significantly higher photoprotective non-photochemical quenching (Y(NPQ)) (Figure 2).

### 3.2. Carbohydrates

Our analysis identified two forms of free monosaccharides (glucose and fructose), two sugar alcohol derivatives (inositol and glycerol), and seven forms of free oligosaccharides (trehalose (in trace amounts), maltose, sucrose, raffinose, stachyose, 1-kestose, and 1,1-tetrakestose).

**Figure 3:**
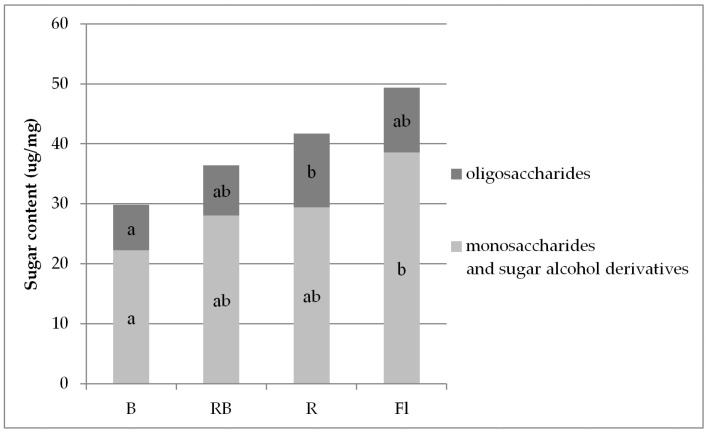
The content of free sugars in the tissues of gerbera multiplied in vitro under light of different quality: B—100% blue LED light (430 nm), RB—a mixture of red and blue LED light (7:3), R—100% red LED light (670 nm), Fl—fluorescent lamps (Philips TL-D 36W/54). The same letters in the chart columns (a, b, ab) indicate means that are not significantly different according to Duncan’s multiple range test at *p* ≤ 0.05.

The levels of these compounds in multiplied gerbera shoots depended on light quality (Figure 3). The mean total content of monosaccharides was three times higher than that of oligosaccharides. The highest mean content of all free carbohydrates was found in the plants multiplied under standard Fl lamps (mean 49.38 µg/mg), and the lowest (by approx. 30% as compared with the highest) under B LED (29.82 µg/mg) (Figure 3, Table S1). The described tendency involved monosaccharides. As for free oligosaccharides, their highest content was determined in the plants growing under R LED (mean 12.29 µg/mg).

**Figure 4:**
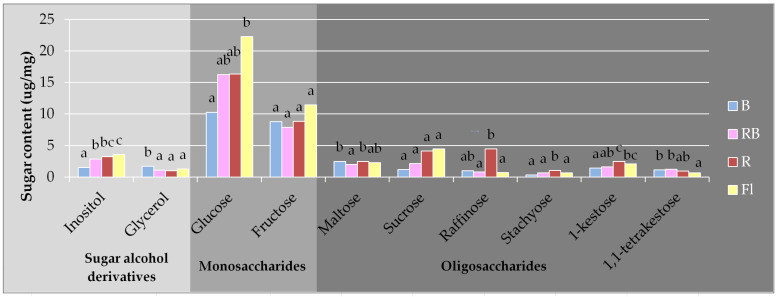
The content of individual identified free sugars in the tissues of gerbera multiplied in vitro under light of different qualities: B—100% blue LED light (430 nm), RB—a mixture of red and blue LED light (7:3), R—100% red LED light (670 nm), Fl—fluorescent lamps (Philips TL-D 36W/54). The same letters above the chart columns (a, b, c, ab, bc) indicate means that are not significantly different according to Duncan’s multiple range test at *p* ≤ 0.05.

The prevailing forms of all identified free sugars were glucose (mean 10.25–22.27 µg/mg) and fructose (7.84–11.42 µg/mg) (Figure 4, Table S2). The content of the remaining free sugars was lower and reached 0.33–4.46 µg/mg. R LED promoted accumulation of free glucose but its content under B LED was on average approximately 44% lower than it was under the other investigated light spectra. Glucose was the most abundant in the plants multiplied under control light emitted by Fl lamps. This trend included a total content of glucose in the pool of soluble sugars (Table 1). The quality of light did not affect the content of free fructose and sucrose in gerbera tissues. B LED reduced the content of inositol and 1-kestose in gerbera tissues. However, plants grown under this light contained more glycerol (B LED) and 1,1-tetrakestose (B and RB LED). R or B LED increased the tissue content of free maltose and raffinose in comparison with that of the mixed spectrum (RB LED). R LED also enhanced the levels of stachyose (Figure 4).

The applied light spectra did not affect the polymerization degree of soluble oligosaccharides (which remained at the average level of 4.21) (Table S3), the content of fructose in the total pool of soluble sugars (Table 1), or the content of starch (on average 0.72 μg/mg) (Table S4).

## 4. Discussion

The most advantageous light spectrum for *G. jamesonii* ‘Big Apple’ seemed to be RB LED light, with a 7:3 red-to-blue ratio. Under these conditions the efficiency of PS II remained high (Y (II)), non-photochemical quenching was the most efficient (Y (NPQ)), and PS II damage was almost as small as under Fl lamps (Y (NO)). The lowest efficiency of PS II (Y (II)) and the greatest damage (Y (NO)) were observed in the plants multiplied under R LED. These effects were associated with the highest level of soluble oligosaccharides. Plants grown under low-energy R LED, despite similar contents of photosynthetic pigments as in other treatments [9], exhibited significantly lower performance of the photosynthetic apparatus, both on the donor (Fv/Fo) and acceptor (Fv/2) side of RC PSII. Abnormalities in the efficiency of OEC activity as well as in the size of the plastoquinone pool resulted in reduced efficiency of PSII (Y(II)) that led to permanent damage of the photosynthetic apparatus (Y(NO)). A similar effect was observed when the photosynthetic apparatus formation was disturbed in plants subjected to various abiotic stressors limiting the synthesis pathways of structural elements of the photosynthetic apparatus [33,43,48,53]. The application of B and RB LED resulted in no significant differences in the photosynthetic apparatus response, which indicated optimal conditions for its formation in gerbera. In conclusion, of the light conditions used, R LED, with the lowest energy of light quanta, appeared to significantly limit the performance of the gerbera photosynthetic apparatus. Yang et al. [54] also reported that different qualities of monochromatic LEDs inhibited plant growth by reducing the activity of the photosynthetic apparatus, with the best results achieved for white light. This may lead to an imbalance in energy distribution between PS II and PS I, and further to growth inhibition [54,55]. The lower energy of R LED may also be connected with the intensity of the light factor, which is very important in the development of the photosynthetic apparatus. *Arabidopsis thaliana* cultivated under low light intensity (25 µmol m^−2^ s^−1^) exhibited significantly lower photosynthetic apparatus efficiency, while plants under moderate light intensity developed photosynthetic apparatuses with the highest efficiency. In contrast, high light intensity (250 µmol m^−2^ s^−1^) led to photoinhibition of PSII as a secondary stress effect [24]. In our study, the intensity of light was constant, while the energy of the light quanta varied considerably.

Plant photosynthetic activity may be limited by sugar addition to an in vitro growth medium [29,56] and also by the quality of light that affects endogenous carbohydrate metabolism. Analyses of sugar levels in in vitro grown gerbera shoots may indicate participation of these compounds in signaling in addition to metabolic pathways. The carbohydrate signaling system, photomorphogenesis, and regulation mechanisms coordinate the supply and consumption of carbon and determine the direction of plant development [57,58]. A high content of sugar may make plants look sturdy and cause their tissues to be thicker. In our study, the greatest amounts of sugar were accumulated in the shoots multiplied under Fl light and the lowest in those grown under B LED. Our previous research showed that the tallest shoots were grown under R and RB LED, which suggests that the shoots grown under fluorescent lamps are thicker as they contain more sugars. However, further analysis indicated low DW of these shoots, while those under RB LED achieved high DW, which was most likely related to their high water content [11,12].

Establishing the composition of low molecular weight sugars is important for characterizing plant physiological and biochemical processes. Monosaccharides are the basic building blocks of saccharides and a common source of energy for metabolism [59]. Our research confirmed high levels of glucose and fructose in gerbera tissues as compared with other identified carbohydrates. This trend was independent of the light quality, both in control and under energy-saving LEDs. The level of glucose was lower only under B LED but when blue light was combined with red (RB LED) this was not observed. Glucose and fructose are signal metabolites and may affect gene expression [57]. Together with numerous bioactive molecules they form part of a complex network and induce different mechanisms independently or via interaction. Changes in glucose content are reflected in plant growth dynamics. The glucose-to-sucrose ratio controls morphogenesis by controlling the rate of cell divisions [60,61]. In studies carried out on a bulb plant, lachenalia, the highest levels of glucose and fructose were found in plants grown under monochromatic red light [62]. Glucose can also tune the plant heterotrophic-to-autotrophic transition and modulate early seedling growth [63].

When gerbera tissues contained more monosaccharides, mainly glucose, as observed under control light, the photosynthetic apparatus performed more efficiently (Y (II)). Although its activity under B LED was also high, it was accompanied by the lowest level of monosaccharides and glucose. B LED was also associated with the lowest efficiency of non-photochemical excitation quenching (Y (NPQ)).

Apart from light, plant growth is also controlled by gravity. Inositol is a sugar-derived element of the signaling pathway engaged in early signaling events in plants, which combine gravity detection with initiation of a gravitropic response [64]. Our study demonstrated an inositol decrease in gerbera tissues under 100% blue light. Previous studies indicated that plants growing under B LED showed poorer growth and lower height [11,12]. Another element of the plant signaling system is glycerol, which, according to the studies of Venugopal et al. [65], may even (along with its metabolites) participate in various physiopathological processes.

Oligosaccharides play structural and/or enzymatic roles and act as signaling molecules. Some of them are also elicitors, i.e., they induce plant defense responses. Our study identified three times lower levels of free oligosaccharides vs. monosaccharides, and R LED stimulated oligosaccharide accumulation. Li et al. [66] reported the highest content of free carbohydrates in *Gossypium hirsutum* L. grown under red light. In contrast to that, Yang et al. [54] found a considerable increase in total soluble sugars in *Nicotiana tabacum* cultivated under B LED. Similarly, Wang et al. [67] and Heo et al. [68] detected the highest content of soluble sugars in the seedlings of *Cucumis sativus* and the highest levels of reducing sugars in grapes grown under blue light. This may be caused by higher activity of the Calvin cycle enzymes that depends on light quality [54]. In our study, R LED enhanced the content of stachyose, which is an important transport carbohydrate [69]. Narrow spectrum light, R LED and B LED, stimulated the production of raffinose. Oligosaccharides from the raffinose family are synthesized from sucrose and also act as antioxidants. They are a part of the carbon partitioning strategies and may serve as stress response signals by stabilizing and mediating stress tolerance [70,71,72,73]. They can also act as reserve carbohydrates and membrane stabilizers [69].

Photosynthetic assimilation of carbon occurs only in light, while growth processes requiring carbon take place throughout the day and night cycles [57,74,75,76,77]. Sucrose can be produced by plants for export and stored in leaf cell vacuoles during the day to provide carbon for further growth overnight [57,78]. In our study, the quality of light did not affect the amount of sucrose or starch accumulated in gerbera tissues. Plant genotypes differ in their starch management, but in general plants of the quickest growth accumulate starch in smaller amounts [57,79]. There are some reports on more intense starch accumulation under red light, e.g., in rape [80] and cotton [66]. In fructan synthesis, sucrose is a donor, while 1-kestose acts as an acceptor [81]. In our study, the latter was the most abundant in the shoots under R LED and Fl. A reverse tendency was observed for 1,1-tetrakestose, the greatest amounts of which were identified under B and RB LED. Maltose, a transport sugar, can be produced by starch degradation and exported from the chloroplast to the cytosol [57,82,83]. Its level in gerbera tissues increased under single wavelength spectra of R or B LEDs. Enhancing the amount of maltose added to the medium may positively affect some specific processes in vitro. Maltose improved development of anther culture in petunia and tomato and affected callus regeneration and somatic embryogenesis of rice [84,85,86,87,88].

## 5. Conclusions

The use of LEDs during clonal reproduction of gerbera did not reduce secondary metabolism and allowed for the production of good quality shoots. Although R LED reduced the efficiency of the photosynthetic apparatus and led to its permanent damage, this was not observed under RB LED (7:3).

The shoots propagated under LED light had the same monosaccharide-to-oligosaccharide ratio as under the fluorescent lamp, i.e., monosaccharides were three times more abundant. Sugars were the most abundant in the shoots multiplied under Fl lamps, and their content under RB LED was moderate. Only the shoots grown under B LED exhibited a reduced sugar content, which was due to a lower glucose level. Glucose in LED-treated plants was the most abundant under mixed RB spectrum.

In comparison with the Fl lamps, RB LED did not affect the levels of free oligosaccharides (including maltose, sucrose, raffinose, stachyose, 1-kestose), monosaccharides (fructose, glucose), or glycerol in in vitro multiplied gerbera shoots. However, it enhanced the content of 1,1-tetrakestose and ensured the most efficient non-photochemical quenching of the PS II excitation state.

To obtain the best quality parameters of the propagated gerbera shoots, we suggest using an RB LED (in 7:3 proportion) that will make in vitro production more environmentally friendly and cost effective but at the same time will enable maintenance of the proper physiological and metabolic performances of the multiplied plants.

## Figures and Tables

**Table 1:** Total content of free glucose and fructose in the pool of soluble sugars (µg/mg) in the tissues of gerbera multiplied in vitro under light of different qualities.

Light Quality	Glucose	Fructose
B ^1^	39.83 ± 7.29 a ^2^	20.20 ± 3.65 a
RB	56.88 ± 11.84 ab	22.39 ± 2.99 a
R	56.84 ± 5.46 ab	24.01 ± 6.30 a
Fl	74.95 ± 21.78 b	32.93 ± 15.89 a

^1^ B—100% blue LED (430 nm); RB—a mixture of red (70%) and blue (30%) LED; R—100% red LED (670 nm); Fl-control, fluorescence (Philips TL-D 36W/54 lamps). ^2^ Means ± standard deviations within a column followed by the same letter (a, b, ab) are not significantly different according to Duncan’s multiple range test at *p* ≤ 0.05.

## Data Availability

Data are contained within the article and Supplementary Materials.

## Supplementary Materials

The following are available online at https://www.mdpi.com/article/10.3390/biology10101035/s1, Table S1: Mean content of free sugars in the tissues of gerbera multiplied in vitro under light of different qualities (µg/mg), Table S2: The content of individual identified free sugars in the tissues of gerbera multiplied in vitro under light of different qualities (µg/mg), Table S3: Degree of polymerization (DP) of soluble oligosaccharides in gerbera axillary shoots multiplied in vitro under different light qualities, Table S4: Content of starch in gerbera axillary shoots multiplied in vitro under different light qualities (µg/mg).
