# Supplementary material for: Energy-Saving LED Light Affects the Efficiency of the Photosynthetic Apparatus and Carbohydrate Content in Gerbera jamesonii Bolus ex Hook. f. Axillary Shoots Multiplied In Vitro
Source: Biology (Basel). 2021 Oct 12;10(10):1035. doi: 10.3390/biology10101035 (PMC8533489; doi:10.3390/biology10101035)
Supplement: Supplementary file 1 [file biology-10-01035-s001.zip › Table S1_v2.pdf]

Table S1. Mean content of free sugars in the tissues of gerbera multiplied *in vitro* under light of different qualities (μg/mg).

**Light quality**	**Monosaccharides and sugar alcohol derivatives**	**Oligosaccharides**	**Σ**
B [1]	22.26 ± 2.04 a [2]	7.56 ± 1.63 a	29.82 ± 2.14 a
RB	28.04 ± 4.19 ab	8.41 ± 2.01 ab	36.45 ± 5.63 ab
R	29.40 ± 6.19 ab	12.29 ± 0.74 b	41.68 ± 6.87 ab
Fl	38.54 ± 11.72 b	10.83 ± 3.36 ab	49.38 ± 14.74 b

[1] B—100% blue LED (430 nm); RB—a mixture of red (70%) and blue (30%) LED; R—100% red LED (670 nm); Fl—control, fluorescence Philips TL-D 36W/54 lamps. [2] Means ± standard deviations within a column followed by the same letter are not significantly different according to Duncan's multiple range test at $p \leq 0.05$.
