# Supplementary material for: Energy-Saving LED Light Affects the Efficiency of the Photosynthetic Apparatus and Carbohydrate Content in Gerbera jamesonii Bolus ex Hook. f. Axillary Shoots Multiplied In Vitro
Source: Biology (Basel). 2021 Oct 12;10(10):1035. doi: 10.3390/biology10101035 (PMC8533489; doi:10.3390/biology10101035)
Supplement: Supplementary file 1 [file biology-10-01035-s001.zip › Table S2_v2.pdf]

Table S2. The content of individual identified free sugars in the tissues of gerbera multiplied *in vitro* under light of different qualities (µg/mg).

Light quality	Sugar alcohol derivatives		Monosaccharides		Oligosaccharides					
	**Inositol**	**Glycerol**	**Glucose**	**Fructose**	**Maltose**	**Sucrose**	**Raffinose**	**Stachyose**	**1-kestose**	**1.1-tetrakestose**
B [1]	1.53 ± 0.13 a [2]	1.70 ± 0.20 b	10.25 ± 1.21 a	8.78 ± 1.09 a	2.49 ± 0.28 b	1.18 ± 1.07 a	1.01 ± 0.21 ab	0.33 ± 0.08 a	1.42 ± 0.13 a	1.13 ± 0.20 b
RB	2.84 ± 0.38 b	1.11 ± 0.25 a	16.26 ± 3.82 ab	7.84 ± 0.49 a	1.98 ± 0.20 a	2.16 ± 1.47 a	0.79 ± 0.10 a	0.64 ± 0.13 a	1.60 ± 0.30 ab	1.23 ± 0.06 b
R	3.24 ± 0.36 bc	1.01 ± 0.22 a	16.31 ± 2.87 ab	8.83 ± 2.83 a	2.48 ± 0.06 b	4.12 ± 0.94 a	4.46 ± 0.06 b	1.02 ± 0.25 b	2.45 ± 0.15 c	0.94 ± 0.20 ab
Fl	3.58 ± 0.24 c	1.28 ± 0.19 a	22.27 ± 6.24 b	11.42 ± 5.40 a	2.26 ± 0.17 ab	4.46 ± 3.22 a	0.71 ± 0.30 a	0.66 ± 0.16 a	2.07 ± 0.47 bc	0.67 ± 0.17 a

[1] B—100% blue LED (430 nm); RB—a mixture of red (70%) and blue (30%) LED; R—100% red LED (670 nm); Fl—control, fluorescence Philips TK-D 36W/54 lamps. [2] Means ± standard deviations within a column followed by the same letter are not significantly different according to Duncan's multiple range test at $p \leq 0.05$.
