# Supplementary material for: Energy-Saving LED Light Affects the Efficiency of the Photosynthetic Apparatus and Carbohydrate Content in Gerbera jamesonii Bolus ex Hook. f. Axillary Shoots Multiplied In Vitro
Source: Biology (Basel). 2021 Oct 12;10(10):1035. doi: 10.3390/biology10101035 (PMC8533489; doi:10.3390/biology10101035)
Supplement: Supplementary file 1 [file biology-10-01035-s001.zip › Table S3_v2.pdf]

Table S3. Degree of polymerization (DP) of soluble oligosaccharides in gerbera axillary shoots multiplied *in vitro* under different light qualities.

Light quality	DP
B [1]	4.01 ± 0.64 a [2]
RB	4.20 ± 0.08 a
R	4.71 ± 1.45 a
Fl	3.92 ± 0.55 a

[1] B—100% blue LED (430 nm); RB—a mixture of red (70%) and blue (30%) LED; R—100% red LED (670 nm); Fl—control, fluorescence Philips TK-D 36W/54 lamps. [2] Means ± standard deviations within a column followed by the same letter are not significantly different according to Duncan's multiple range test at $p \leq 0.05$.
