# Supplementary material for: Energy-Saving LED Light Affects the Efficiency of the Photosynthetic Apparatus and Carbohydrate Content in Gerbera jamesonii Bolus ex Hook. f. Axillary Shoots Multiplied In Vitro
Source: Biology (Basel). 2021 Oct 12;10(10):1035. doi: 10.3390/biology10101035 (PMC8533489; doi:10.3390/biology10101035)
Supplement: Supplementary file 1 [file biology-10-01035-s001.zip › Table S4_v2.pdf]

Table S4. Content of starch in gerbera axillary shoots multiplied *in vitro* under different light qualities (µg/mg).

Light quality	Starch
B [1]	0.69 ± 0.06 a [2]
RB	0.68 ± 0.26 a
R	0.73 ± 0.11 a
Fl	0.78 ± 0.34 a

[1] B - 100% blue LED light (430 nm); RB—a mixture of red (70%) and blue (30%) LED light; R - 100% red LED light (670 nm); Fl - control, fluorescence Philips TK-D 36W/54 lamps. [2] Means ± standard deviations within a column followed by the same letter are not significantly different according to Duncan's multiple range test at $p \leq 0.05$
